# Characterization of Mesenchymal Stem Cell-Like Cells Derived From Human iPSCs via Neural Crest Development and Their Application for Osteochondral Repair

**DOI:** 10.1155/2017/1960965

**Published:** 2017-05-10

**Authors:** Ryota Chijimatsu, Makoto Ikeya, Yukihiko Yasui, Yasutoshi Ikeda, Kosuke Ebina, Yu Moriguchi, Kazunori Shimomura, David A Hart, Yoshikawa Hideki, Nakamura Norimasa

**Affiliations:** ^1^Graduate School of Medicine, Orthopaedic Surgery, Osaka University, Suita, Osaka, Japan; ^2^Graduate School of Frontier Bio Science, Orthopaedic Surgery, Osaka University, Suita, Osaka, Japan; ^3^Center for iPS Cell Research and Application, Life Science Frontiers, Kyota University, Kyoto, Kyoto, Japan; ^4^Graduate School of Medicine, Orthopaedic Surgery, Sapporo Medical University, Sapporo, Hokkaido, Japan; ^5^McCaig Institute for Bone and Joint Health, University of Calgary, Calgary, Canada; ^6^Institute for Medical Science in Sports, Osaka Health Science University, Osaka, Osaka, Japan; ^7^Grobal Center for Medical Engineering and Informatics, Osaka University, Suita, Osaka, Japan

## Abstract

Mesenchymal stem cells (MSCs) derived from induced pluripotent stem cells (iPSCs) are a promising cell source for the repair of skeletal disorders. Recently, neural crest cells (NCCs) were reported to be effective for inducing mesenchymal progenitors, which have potential to differentiate into osteochondral lineages. Our aim was to investigate the feasibility of MSC-like cells originated from iPSCs via NCCs for osteochondral repair. Initially, MSC-like cells derived from iPSC-NCCs (iNCCs) were generated and characterized in vitro. These iNCC-derived MSC-like cells (iNCMSCs) exhibited a homogenous population and potential for osteochondral differentiation. No upregulation of pluripotent markers was detected during culture. Second, we implanted iNCMSC-derived tissue-engineered constructs into rat osteochondral defects without any preinduction for specific differentiation lineages. The implanted cells remained alive at the implanted site, whereas they failed to repair the defects, with only scarce development of osteochondral tissue in vivo. With regard to tumorigenesis, the implanted cells gradually disappeared and no malignant cells were detected throughout the 2-month follow-up. While this study did not show that iNCMSCs have efficacy for repair of osteochondral defects when implanted under undifferentiated conditions, iNCMSCs exhibited good chondrogenic potential in vitro under appropriate conditions. With further optimization, iNCMSCs may be a new source for tissue engineering of cartilage.

## 1. Introduction

Cartilage injuries usually do not heal spontaneously; therefore, various cell therapies using chondrocytes or mesenchymal stem cells (MSCs) have been investigated to overcome the clinically poor outcomes [1]. Among them, chondrocyte-based therapies have been extensively examined since the initial reports of successful autologous chondrocyte implantation (ACI). However, there are potential concerns regarding the limited availability of chondrocytes due to the limited size of harvested intact cartilage, and dedifferentiation of the chondrocytic phenotype associated with in vitro monolayer expansion. In this regard, MSCs derived from bone marrow, adipose tissue, and other connective tissues are promising alternatives for cartilage repair because of their expandability and chondrogenic potential. However, these cell sources also likely have some limitations, including limited cell proliferative capacity and loss of cell viability during long-term culture [2]. Moreover, the quality of MSCs varies widely among donors [3–5]; therefore, MSC therapies are not always available to all the patients.

Pluripotent stem cells (PSCs), such as embryonic stem cells (ESCs) and induced pluripotent stem cells (iPSCs), could be alternative sources for various cell therapies including cartilage repair. PSCs exhibit infinite growth and differentiation and can be obtained with minimally [6] or less invasive procedures [7]; however, the safety for clinical usage of PSCs and their derivatives has not been fully elucidated [8]. Also, the instability of the phenotype associated with differentiation protocols remains to be resolved.

Previous studies reported methods to produce MSC-like cells from human PSCs for targeting bone and/or cartilage repair [9–15]. The generation of intermediate, MSC-like cells between PSCs and terminally differentiated cells could be a promising strategy for the purification of osteogenic/chondrogenic progenitors and the elimination of residual PSCs. In many of the early studies, MSC-like cells were directly induced from PSCs by serum-containing medium without any signal control. Outgrowth cells from PSC colony or embryoid body cultured with such medium were collected based on their proliferative potential, and those cells have been known to satisfy the criteria of in vitro MSC [11, 12, 14, 16–21]. There have been some studies outlining how to prepare the PSCs prior to the induction of MSCs, when to switch to MSC medium from PSC medium, and how to expand those induced cells. However, such MSC-like cells, induced by only serum-containing medium, have decreased differentiation potential, particularly toward the chondrogenic lineage [15, 19, 22–29]. Therefore, adequate signal control, mimicking embryonic development, is considered a necessity to create useful cells for cartilage repair.

Recently, neural crest cells (NCCs) have been reported as an effective pathway to induce mesenchymal progenitors, as the resulting cells appear to have high potential for differentiation into osteocytes and chondrocytes [30–33]. NCCs are known to give rise to many cranial tissues including bone and cartilage [34–36], but NCC-derived cells have been detected in the bone marrow of limb tubular bones [37–39]. These cells obtained proliferative and multipotent differentiation properties after in vitro culture; therefore, the neural crest is considered as one of the origins of MSCs [39–42].

For the study of PSCs, simple and efficient methods to generate NCCs from iPSCs (iNCCs) have already been established by several groups [30–32, 43–46]. The activation of canonical Wnt signaling and the prevention of TGF*β* signaling are common approaches in obtaining a highly enriched population of CD271^(+)^-iNCCs. Most remarkably and beneficially, iNCCs can be expanded for long term under conditions of bFGF supplementation and TGF*β* inhibition; moreover, frozen stocks can be made [30, 31, 43], which could be a great advantage for future clinical use. iNCCs have the potential to differentiate into peripheral neurons, glia, pigment cells, corneal endothelial cells, and other cell types [44, 45], but not directly to osteocytes or chondrocytes. Therefore, it is likely necessary to first switch to the mesenchymal lineage, as shown in previous reports [12, 21, 47–49].

Although it is known that PSC-derived NCCs have osteogenic and chondrogenic potential in vitro after mesenchymal induction [30, 32, 33, 43, 50, 51], there have been no further published research regarding the use of iNCC-derived MSC-like cells (iNCMSCs) for bone or cartilage repair in vivo. Thus, the purpose of the present study was to generate and characterize MSC-like cells from iNCCs and, subsequently, to investigate their capacity to repair cartilage and bone in vivo using an athymic nude rat osteochondral defect model.

## 2. Materials and Methods

### 2.1. Cell Culture

Human NCCs derived from 414C2-iPSCs (iNCCs) were a kind gift from Dr. M. Ikeya and were maintained as described previously [30]. In brief, iNCCs were cultured on fibronectin (Millipore, Bedford, CA, USA) coated dishes and maintained with chemically defined medium (CDM), which contains Iscove's modified Dulbecco's medium/Ham's F-12 1 : 1, 1× chemically defined lipid concentrate (Gibco, Grand Island, NY, USA), 15 *μ*g/mL apo-transferrin (Sigma-Aldrich, St. Louis, MO, USA), 450 mM monothioglycerol (Sigma-Aldrich), 5 mg/mL purified BSA (Sigma-Aldrich), 7 *μ*g/mL insulin (WAKO, Osaka, Japan), 10 *μ*M SB431542 (SB) (Selleck Chemicals, Houston, Texas, USA), 20 ng/mL EGF (R&D Systems, Minnesota, USA), 20 ng/mL bFGF (Wako), and penicillin/streptomycin (Invitrogen, Carlsbad, CA, USA). iNCCs were passaged with Accutase (Gibco) and replated at 1 × 10^4^ cells/cm^2^. The medium was replaced every 2-3 days.

For MSC induction from iNCCs, the CDM was replaced with MSC medium, which contained alpha-MEM (aMEM) (Nakarai Tesque, Tokyo, Japan), 10% fetal bovine serum (FBS) (Sigma-Aldrich), 5 ng/mL bFGF, and 1% antibiotic antimycotic (Sigma-Aldrich). The next day, cells were harvested with 0.25% Trypsin-EDTA (Gibco) and replated onto tissue culture dishes (Corning, NY, USA) at a density of 1–3 × 10^3^ cells/cm^2^. The MSC medium was changed twice a week, and cells were passaged with 0.25% Trypasin-EDTA at 80–90% confluency. All cells were incubated at 37°C with 5% CO_2_. The culture conditions for other cells are described in the Supplementary Methods available online at https://doi.org/10.1155/2017/1960965.

Senescence-associated *β*-galactosidase staining was performed using a Cellular Senescence detection kit (Cell Biolabs, San Diego, California, USA). Population doubling level (PDL) was calculated using the formula PDL = log(*N*/*N*0)/log2, where *N*0 is the plated cell number and *N* is the harvested cell number at the time of passage. Cumulative PDL was the total PDL = Σ(PDL)*n*, where *n* is the passage number [52]. Population doubling time (PDT) was calculated using the formula PDT = culture period (hours)/PDL [53].

Human bone marrow-derived MSC were obtained from LONZA (catalog number PT-2501, lot number 0000451491) and used for assays after three passages with the same MSC medium as described above.

### 2.2. Fluorescence-Activated Cell Sorting (FACS)

FACS was performed using BD FACSVerse™ flow cytometer (BD Bioscience, New Jersey, USA) according to the manufacturer's protocol. iNCCs and iNCMSCs were dissociated into single cells as described above. Cells were resuspended in 0.1%BSA-PBS and incubated for 30 min at room temperature with fluorescence-conjugated antibodies. The antibodies used for FACS are listed in Supplementary Table S1. Appropriate antibodies were used as a negative control. The cells were washed with 0.1%BSA-PBS twice and then suspended in 0.5 mL of 0.1%BSA-PBS for analysis with a BD FACSVerse (BD Biosciences). Data retrieved from the sorting was analyzed with BD FACSuite Software (BD Bioscience).

### 2.3. Differentiation of iNCMSCs

Cells from passages 2-3 were used for the following in vitro differentiation assays for chondrogenesis, osteogenesis, and adipogenesis.

#### 2.3.1. Induction of Chondrogenesis

To obtain cell aggregated pellets, 2 × 10^5^ cells were centrifuged in a 96-deep well polypropylene plates and cultured with 10% FBS-aMEM. The next day, the medium was changed to a chondrogenic medium that consisted of high-glucose DMEM containing 110 *μ*g/mL sodium pyruvate (Gibco) supplemented with 0.2 mM ascorbate-2-phosphate (Sigma-Aldrich), 40 *μ*g/mL L-proline (Wako), 100 nM dexamethasone (Sigma-Aldrich), 1% ITS + Premix (Corning: 6.25 *μ*g/mL insulin, 6.25 *μ*g/mL transferrin, 6.25 *μ*g/mL selenious acid, 1.25 *μ*g/mL bovine serum albumin, and 5.35 *μ*g/mL linoleic acid), 10 ng/mL transforming growth factor-*β*3 (TGF*β*3) (Peprotech, Rocky Hill, New Jersey, USA), and 50 ng/mL bone morphogenic protein 2 (BMP2) (Medtronic, Dublin, Ireland). The pellets were maintained with 0.5 mL of chondrogenic culture medium in a humid atmosphere of 5% CO_2_ at 37°C. The medium was replaced twice per week.

Total sulfated glycosaminoglycan (sGAG) was measured using a Blyscan Assay Kit (Biocolor, Westbury, NY, USA) based on 1,9-dimethylmethylene blue binding against a standard curve of chondroitin-6-sulfate according to the manufacturer's protocol.

#### 2.3.2. Induction of Osteogenesis

iNCMSCs were seeded at a density of 5 × 10^4^/cm^2^ and cultured in osteogenesis differentiation medium (Gibco). Cells cultured in 10%FBS-aMEM served as a control. Differentiation was confirmed by assessing alkaline phosphatase (ALP) activity and calcium deposition. For analysis, cells were rinsed twice with PBS, fixed with 4% paraformaldehyde (PFA) for 10 minutes, and washed with water. Alkaline phosphatase (ALP) activity was detected using the BCIP/NBT Color Development Substrate (Promega, Wisconsin, USA), according to the manufacturer's instruction. Calcium deposition was detected by 1% alizarin red S solution (pH 6.4) (Muto Pure Chemicals, Tokyo, Japan) for 10 minutes at room temperature and then washed thoroughly with water. For von Kossa staining, culture wells were stained with 5% silver nitrate (Wako) under ambient light for 1 hour and then fixed with 5% sodium thiosulfate (Wako) for 5 min to remove nonreacted silver.

#### 2.3.3. Induction of Adipogenesis

iNCMSCs were seeded at a density of 1 × 10^5^/cm^2^ and cultured in 10%FBS-aMEM until over confluent. Subsequently, the cells were cultured in adipogenesis differentiation medium (Gibco) for 72 hours, followed by incubation in adipogenic maintenance medium (10%FBS-aMEM, 10 *μ*g/mL insulin) for 72 hours. Adipogenesis was induced by cycles of induction/maintenance [54]. Cells cultured in 10%FBS-aMEM served as a control. To detect the formation of lipid vacuoles, cells were fixed with 4% PFA, washed, and stained with oil red O solution (0.5% oil red O (Sigma-Aldrich) in 60% isopropanol) for 15 minutes. The stained dye was eluted with 100% isopropanol, and the absorbance was measured at 520 nm.

### 2.4. Development of the Tissue-Engineered Constructs (TEC)

In order to prepare an in vitro engineered tissue, which is feasible for implantation, the cells from passage 2 were plated in tissue culture vessels at a density of 4.0 × 10^5^ cells/cm^2^ as previously reported [55, 56] and cultured in 10%FBS-aMEM. After 7 days of overconfluent culture, a complex of cultured cells and the ECM synthesized by the cells was detached from the substratum by applying shear stress through using gentle pipetting. The detached monolayer complex was left in suspension to form a three-dimensional structure by active tissue contraction.

### 2.5. Implantation of TEC to Osteochondral Defects

Animal experiments were performed according to a protocol approved by the Animal Care and Use Committee of The University of Osaka. Twelve-week-old male euthymic nude rats (F344 NJcl-rnu/rnu; CLEA Japan, Fujinomiya, Japan) were anesthetized by an intraperitoneal injection of a mixture of 0.3 mg/kg of medetomidine, 4.0 mg/kg of midazolam, and 5.0 mg/kg of butorphanol. For both knees, the femoral trochlear grooves were exposed via a medial parapatellar incision with lateral patellar dislocation. A longitudinal, full-thickness osteochondral defect (1.2 mm wide, 3-4 mm long, and 1 mm deep) was made in each knee by manual drilling, taking care to avoid overheating during the drilling procedure. The TEC that were developed in 24-well plates were implanted into the defects, and then patellar dislocation was reduced. The joint capsule and the skin were sutured in separate layers. After surgery, rats were allowed to be active without any fixation device or immobilization. Animals were euthanized with CO_2_ inhalation at 4 weeks or 8 weeks postoperation. The repaired tissues were scored for macroscopic appearance, based on the following tissue color-grading scheme: 1 = depression (tissue void), 2 = red/grey, 3 = beige, 4 = red-white, and 5 = white, homogeneous tissue [57].

### 2.6. Histology and Histochemistry

The samples were fixed in 4% PFA and embedded in paraffin wax, which was followed by dehydration with serial ethanol and clearance with xylene. For evaluation of the osteochondral tissues, the dissected femoral ends were decalcified with 10% ethylenediaminetetraacetic acid (pH 7.4) before paraffin embedding. The specimens were cut into 5 *μ*m thick sections and used for Safranin O-fast green-iron hematoxylin (Saf-O) staining or immunohistochemistry. The information regarding the antibodies and reaction conditions is listed in Supplemental Table S2. The immune complexes, antigen-first antibody, were detected by Histofine Simple Stain MAX PO (Nichirei Biosciences, Tokyo, Japan) and Simple Stain DAB Solution (Nichirei). Most slides were counterstained with hematoxylin, and some slides were additionally stained with alcian blue (pH 1.0) (Muto Pure Chemicals) [58].

The histological sections of repaired tissues stained with Saf-O were used for evaluation using the modified O'Driscoll score for cartilage and subchondral bone repair [59].

### 2.7. RNA and qRT-PCR

Total RNA was extracted with a RNeasy Mini Kit (QIAGEN, Valencia, CA, USA) for expanded cells and RNeasy Fibrous Tissue Mini Kit (QIAGEN) for chondrogenic pellets. RNase-Free DNase Set (QIAGEN) was used to remove genomic DNA. Total RNA was reverse transcribed into first-strand complementary DNA (cDNA) using Super Script VILO (Life Technologies, Maryland, USA) and random primers, according to the manufacturer's protocol. Quantitative RT-PCR was performed using Taqman assays (Applied Biosystems, California, USA), Taqman Fast Advanced Master Mix (Applied Biosystems), and StepOne Plus (Applied Biosystems). The information of Taqman assays is listed in Supplementary Table S3. Target transcriptional levels were normalized to the level of glyceraldehyde 3-phosphate dehydrogenase (GAPDH) expression. The expression levels of each target genes were represented by the *Δ*ct value (target Ct − GAPDH Ct) or calculated value (2^−*Δ*ct^) [60, 61].

## 3. Results

### 3.1. Induction and Characterization of MSCs from iNCCs (iNCMSCs)

**Figure 1:**
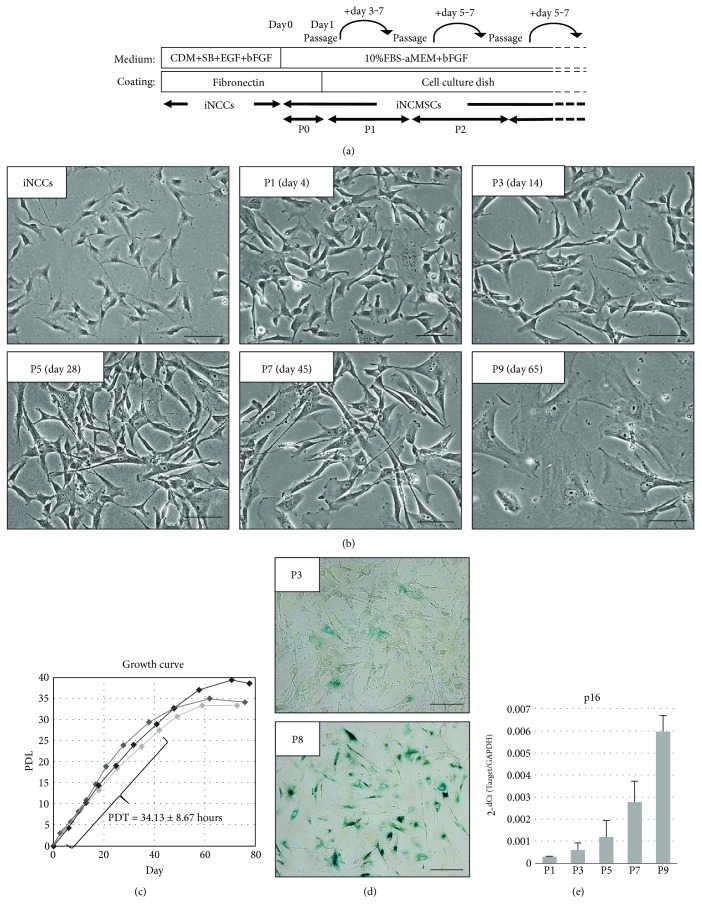
Transition to iNCMSCs from iNCCs and their expandability. (a) Schematic protocol for generation of iNCMSCs from iNCCs. (b) Cell morphology of iNCCs and iNCMSCs during expansion culture. Passage number and expanded days are presented in their inboxes. (c) Growth curve of iNCMSCs over two months (*N* = three lines). Each symbol represents each passage. PDT was calculated during first seven weeks (mean ± SD for three lines). (d) Senescence-associated *β*-galactosidase staining of iNCMSCs at passages 3 and 8. (e) Upregulation of p16 mRNA expression during expansion culture (means ± SD for three lines). Scale bars = 100 *μ*m (b, d). Abbreviations: CDM: Chemical defined medium; SB: SB431452; PDL: Population doubling level; PDT: Population doubling time; iNCCs: Induced neural crest cells; iPSCs: Induced pluripotent stem cells; iNCMSCs: iNCC-derived mesenchymal stem cells.

Provided iNCCs were generated from 414C2-iPSCs through sorting of CD271^high^ cells after 7 days cultivation with chemically defined medium supplemented with 10 *μ*M SB431542 (SB) and 1 *μ*M CHIR99021 [30] and maintained with chemically defined medium supplemented with SB, EGF, and bFGF on fibronectin-coated dish [30]. To derive MSCs from iNCCs, iNCC medium was switched to 10%FBS-aMEM supplemented with 5 ng/mL bFGF. Twenty-four hours later, the cells were passaged with trypsin and subcultured on general cell culture dish (Figure 1(a)). More than six lines of iNCMSCs were generated from 414C2-iNCCs at different timing. Once switched to MSC medium, all lines quickly changed their cell morphology to fibroblast-like spindle cells and displayed homogenous population within a few weeks (Figure 1(b)). The growth was rapid in the first seven weeks: population doubling time (PDT) was approximately 36 hours, and iNCMSCs had high mitotic potential over thirty times (Figure 1(c)), which is comparable to conventional human MSCs [53, 62]. Over eight weeks of culture, their growth slowed down and morphologically enlarged and flat cells were increased in abundance (Figures 1(b) and 1(c)). Such cells showed positive staining for senescence-associated *β*-GAL staining (Figure 1(d)), and moreover, the mRNA expression for p16, which is a marker of cell senescence, was upregulated with increasing passage number (Figure 1(e)). Abnormal propagation and/or spheroid formation, as is observed with tumor cells, was not detected.

**Figure 2:**
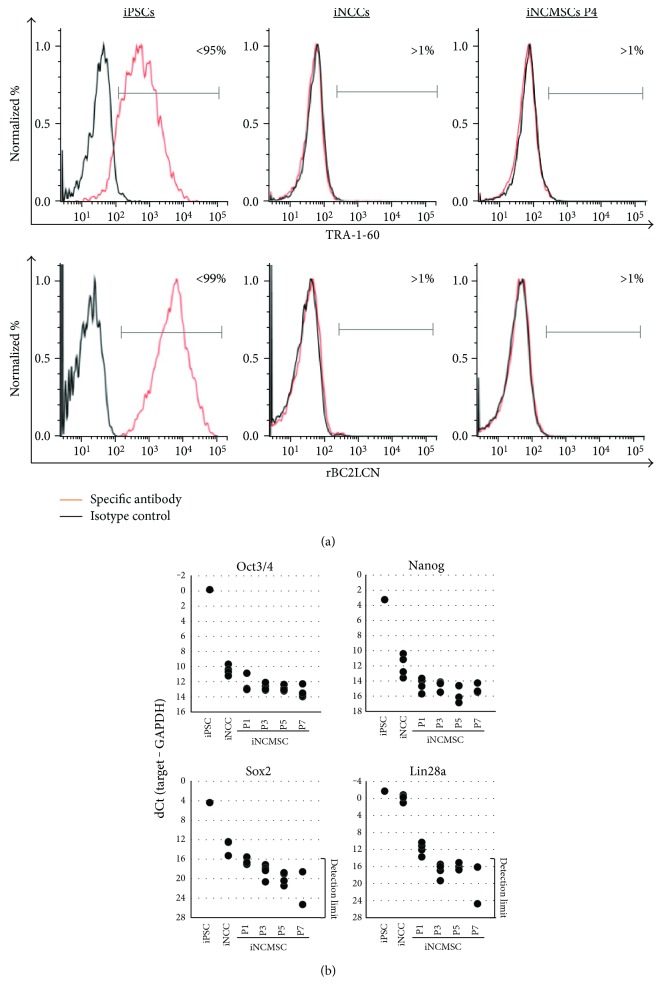
Depletion of pluripotent markers in iNCMSCs. (a, b) FACS analysis of PSC-specific surface antigen and gene expression analysis of PSC-specific transcript factors in iPSCs, iNCCs, and iNCMSCs. 409B2-iPSCs were used for positive control of each assays. Abbreviations: PSCs: Pluripotent stem cells; iNCCs: Induced neural crest cells; iPSCs: Induced pluripotent stem cells; iNCMSCs: iNCC-derived mesenchymal stem cells.

With regard to the pluripotency, iNCMSCs rarely expressed the pluripotent stem cell- (PSC-) specific surface antigens (TRA-1-60 and rBC2LCN) based on FACS analysis (Figure 2(a)), and the PSC-specific transcript factors (Oct4, Nanog, Sox2, and Lin28a) were quickly downregulated to nearly undetectable level during MSC induction (Figure 2(b)).

**Figure 3:**
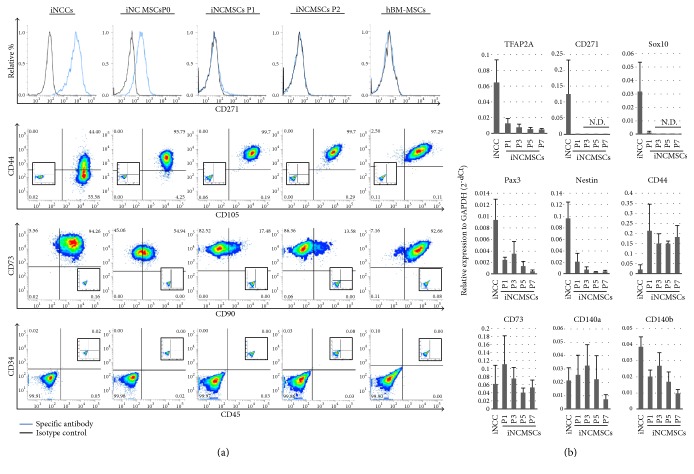
Neural crest markers and mesenchymal stem cell markers. (a) FACS analysis of NC and MSC surface markers in iNCC and iNCMSC (P0, P1, and P2). The date are representative of four lines. Human bone marrow derived MSCs were used for assay control. (b) Gene expression of NC and MSC markers during expansion of iNCMSCs. (mean ± SD for four lines). Abbreviations: iNCCs: Induced neural crest cells; iPSCs: Induced pluripotent stem cells; iNCMSCs: Induced neural crest derived mesenchymal stem cells; hBM-MSCs: Human bone marrow-derived MSCs.

CD271, which is a neural crest marker, was also rapidly lost in response to exposure to the MSC media (Figures 3(a) and 3(b)), and MSC markers such as CD44, CD73, and CD105 also converged to similar expression patterns in passaged iNCMSCs, to levels similar to those for human bone marrow-derived MSCs (hBMMSCs) (Figure 3(a)). The cells retained their cell surface antigen profile until passage 8 (Supplementary Figure S1A). CD34 and CD45, which are hematopoietic stem cell markers, were not detected, similar to findings observed with hBMMSCs (Figure 3(a) and Supplementary Figure S1B). Unexpectedly, the expression of CD90, which is a general MSC marker, was lost with increasing passage number; however, iNCMSCs cultured without bFGF consistently expressed CD90 (Supplementary Figure S2). The mRNA levels for neural crest markers (TFAP2A, CD271, Sox10, Pax3, and nestin) were also downregulated immediately upon MSC induction (Figure 3(b)).

These results suggest that MSC-like cells can be rapidly and homogeneously obtained from neural crest cells during an initial few passages, without detectable contamination by malignant cells or remnant neural crest cells.

### 3.2. Differentiation Potential of iNCMSCs toward Chondrocytes, Osteocytes, and Adipocytes

To investigate the differentiation potential of iNCMSCs for further use in vivo, their abilities to differentiate towards the chondrogenic, osteogenic, and adipogenic lineages were assessed using standard methods known from a number of MSC studies [27, 32, 54, 63].

**Figure 4:**
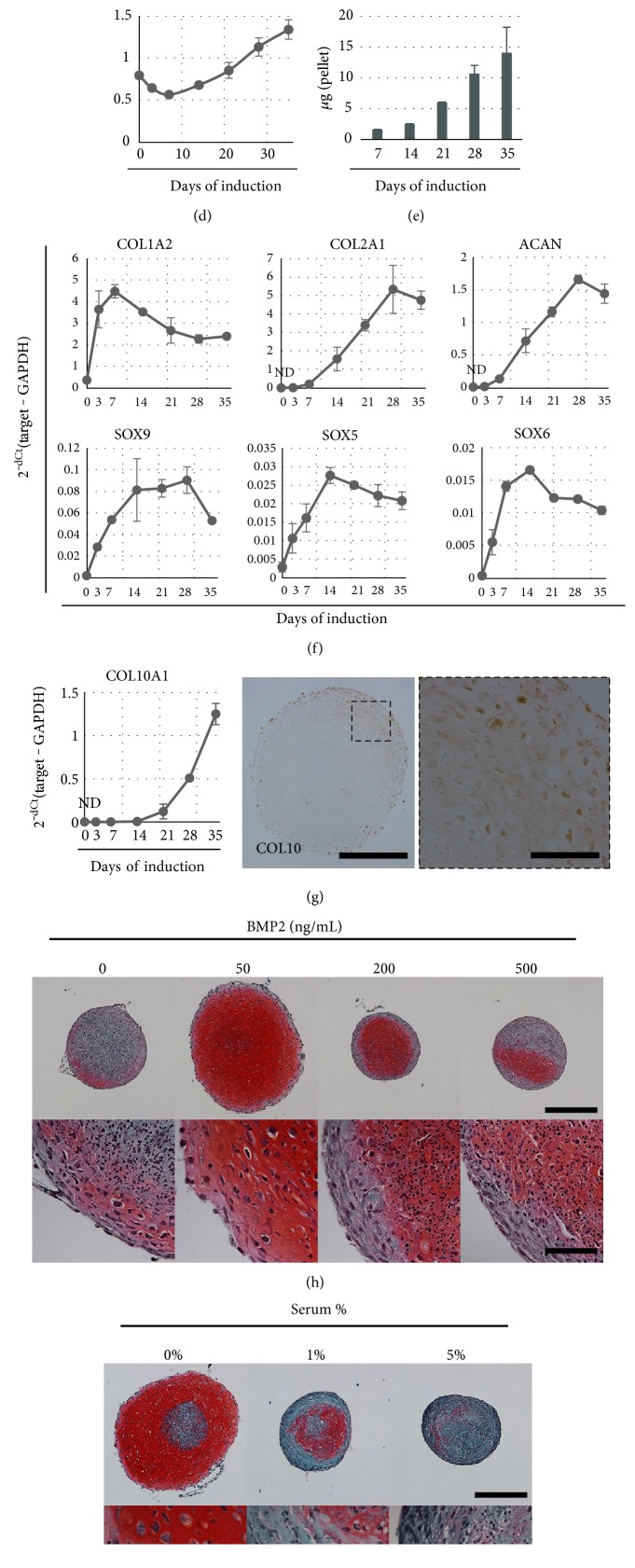
Chondrogenesis of iNCMSCs. The representative data from three lines is shown. (a) Safranin-O staining of chondrogenic pellet stimulated with TGF*β*3 and BMP2 at day 28. High magnification image is shown in the right panel with the same frame. (b, c) Immunostaining for COL2 and SOX9 of chondrogenic pellet at day 28. (d) The pellet size of chondrogenic pellets during culture. (e) Deposition of sulfated glycosaminoglycan in chondrogenic pellets during culture. (f) The alteration of chondrogenesis-related gene expression. (g) mRNA expression levels for type 10 collagen in long-term culture and immunostaining for type 10 collagen at day 28 in chondrogenic pellets. (h) Saf-O images of chondrogenic pellet culture with TGF*β*3 (10 ng/mL) and various concentrations of BMP2 (0–500 ng/mL) for 28 days. (i) Saf-O images of chondrogenic pellet cultures in TB media with addition of variable serum concentrations for 28 days. Scale bars = 500 *μ*m (a, h, i) and 100 *μ*m (b, c, and high-magnified images of a, h, i). Data are expressed as mean ± SD for three pellet replicates (d–g). Abbreviations: Saf-O: Safranin-O staining; COL2: Type 2 collagen; sGAG: Sulfated glycosaminoglycan; COL10: Type 10 collagen.

To assess their chondrogenic potential, iNCMSCs were subjected to a 3D pellet culture system using TGF*β*3 and BMP2. Although stimulation with only TGF*β*3 or BMP2 individually did not induce chondrogenesis, the pellets cultured with TGF*β*3 and BMP2 (TB) underwent chondrogenesis, as indicated by strong stained with Safranin-O (Figure 4(a)). Furthermore, the pellets contained type 2 collagen and most cells expressed SOX9 (Figures 4(b) and 4(c)). The size of the pellet continued to increase in parallel with sulfated glycosaminoglycan (sGAG) deposition (Figures 4(d) and 4(e)). In the assessment of mRNA levels, those for the SOX trio (SOX5, SOX6, and SOX9), transcriptional regulators of chondrogenesis were upregulated by day 3 and reached a peak by day 14. Similarly, those for COL2A1 and ACAN, major matrices of hyaline cartilage were upregulated by day 7 and continued to rise until day 28. In contrast, an upregulation of mRNA levels for COL1A2 was not observed during the culture period (Figure 4(f)). These results suggest that iNCMSCs have potential for chondrogenesis and formation of hyaline-like cartilage tissue. In addition, over the 3-week induction period, exposure to the TB media led to upregulated expression of mRNA for COL10, a marker of hypertrophic chondrocytes, which implied that the endochondral ossification pathway was also activated (Figure 4(g)). Notably, chondrogenesis induction of iNCMSCs could only be induced with an adequate dose of cytokines (Figure 4(h)), and only a small amount of contamination of serum appeared to severely inhibit their chondrogenesis commitment, unlike conventional MSC [64–68]. On the other hand, iNCCs did not aggregate spontaneously in direct chondrogenic pellet culture (data not shown); findings consist with a previous report [31]. Such evidence indicates that transition to a mesenchymal lineage is required for these cells prior to giving rise to cartilage-like differentiation by some stimulation factors.

**Figure 5:**
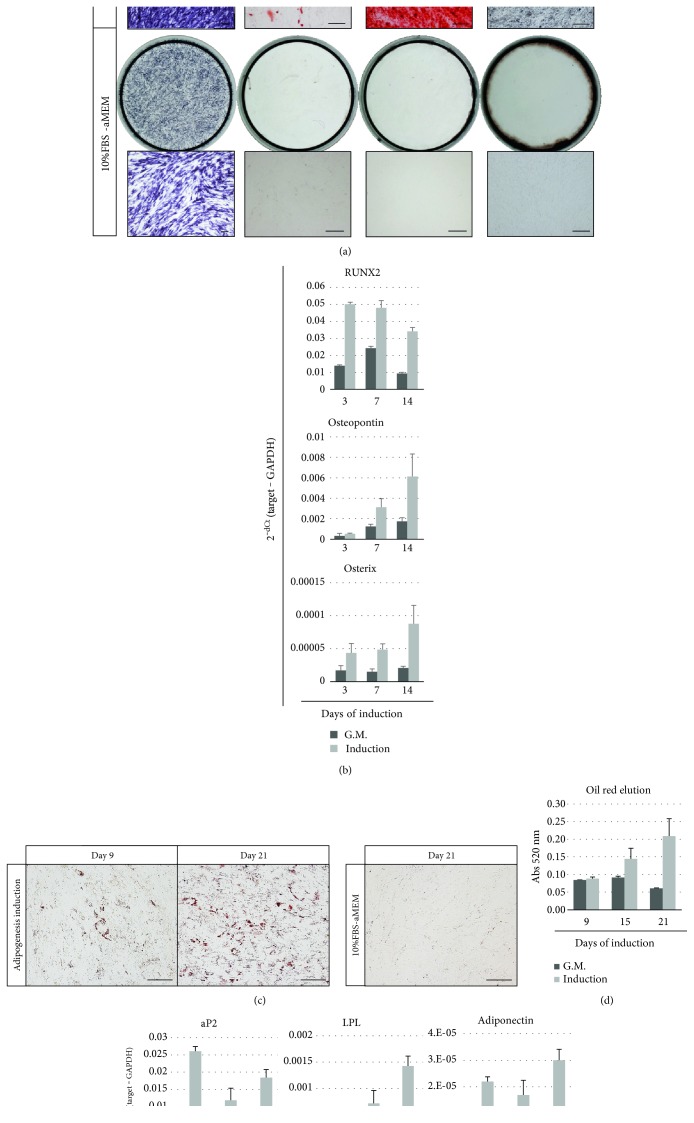
Osteo- and adipogenesis of iNCMSCs. The representative data for three lines is shown. (a) ALP staining at day 7 and calcium staining (alizarin red S and von Kossa staining) at day 14 of iNCMSCs cultured in osteogenic medium in 24-well plates. (b) Osteogenesis-related gene expression at days 3, 7, and 14. (c) Oil red staining of iNCMSCs cultured in adipogenesis medium at days 9 and 21. (d) Measurement of oil red positive droplets. (e) Adipogenesis-related gene expression at day 9, 15, and 21. Data are expressed as mean ± SD for three well replicate per group (b, d, e). Scale bars = 100 *μ*m (a, c). Abbreviations: ALP: Alkaline phosphatase; G.M.: Growth medium.

Osteogenesis and adipogenesis were confirmed with a commercially available differentiation media. As shown in Figure 5(a), iNCMSCs cultured in the osteogenesis differentiation media exhibited high ALP activity compared with those that were maintained in growth medium. Moreover, calcium deposition was confirmed by alizarin red and von Kossa staining. Osteocyte-specific genes such as RUNX2, osteopontin, and osterix were also upregulated through osteogenesis induction (Figure 5(b)).

To induce adipogenesis, iNCMSCs were stimulated with adipogenesis differentiation media. As the results show, the formation of fatty droplets was confirmed (Figures 5(c) and 5(d)), and adipogenesis-specific genes such as those encoding aP2, LPL, and adiponectin were upregulated only in induced cells (Figure 5(e)). However, their adipogenic potential was somewhat inferior to that resulting from differentiation of adult bone marrow-derived MSCs (Supplementary Figure S3).

These results suggested that iNCMSCs have the potential for tri-lineage differentiation, which is known as a property of MSCs. Notably, chondrogenesis and osteogenesis were sufficiently induced, findings which support their potential use for osteochondral repair.

### 3.3. Development of Engineered Tissue from iNCMSCs

There are various approaches to cartilage repair using MSCs. Some studies have simply used undifferentiated MSCs [12, 14, 69, 70], while other studies have induced such cells to commit to the chondrocyte lineage prior to implantation [71–75]. Initially, the feasibility of using iNCMSCs, without prior chondrogenic commitment to repair osteochondral defects in an immunocompromised rat model, was assessed.

**Figure 6:**
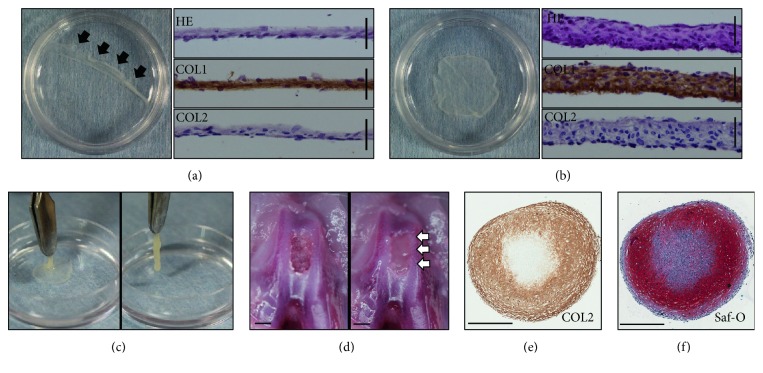
In vitro development of tissue-engineered tissue from iNCMSCs. (a–c)ses gross appearance of iNCMSC-TEC developed in 35 mm dishes at day 7. Thin monolayer cell sheet was formed in the culture bottom surface after 7 days of high-density culture (a), and once artificially detached (black arrows), a thick sheet-like construct (iNCMSC-TEC) developed through active tissue contraction within a few minutes (b). HE staining and immunostaining for COL1 and COL2 of transverse section are shown in their corresponding right panels. Scale bars = 50 *μ*m. The iNCMSC-TEC exhibited sufficient strength to handle with forceps (c). (d) Ex vivo implantation of iNCMSC-TEC into osteochondral defects of cadaver rats. iNCMSC-TEC readily filled the defect created in the femoral groove (white arrows). Scale bars = 1 mm. (e, f) Chondrogenic potential of iNCMSC-TEC. COL2 immunostaining (e) and Safranin-O staining (f) of iNCMSC-TEC cultured in chondrogenic medium for 1 month. Scale bars = 500 *μ*m. Abbreviations: HE: Hematoxylin and eosin staining; COL1: Type 1 collagen; COL2: Type 2 collagen; Saf-O: Safranin-O.

To implant iNCMSCs into osteochondral defects, a scaffold-free tissue-engineered construct- (TEC-) mediated method was adopted [55, 76–80]. This approach enabled the engraftment of numerous stem cells, and the feasibility to repair rodent osteochondral defects has been demonstrated [80]. By seeding and maintaining the cells at a high density, iNCMSCs developed a thin sheet-like structure that adhered to the bottom of the culture vessel (Figure 6(a)). Once detached from the culture vessel by exerting shear stress at the cell/substratum interface, the cell sheet contracted spontaneously and exhibited a thick sheet-like structure (Figure 6(b)). Such iNCMSC-derived constructs (iNCMSC-TEC) could be derived within one week, and they were of sufficient strength to be handled with forceps (Figure 6(c)); therefore, they were readily implanted into osteochondral defects created in the femoral groove of cadaver rat knees (Figure 6(d)). iNCMSC-TEC were initially a fibrous tissue rich in type 1 collagen, but they evolved to be a cartilaginous-like tissue consisting of type 2 collagen following stimulation with TB in vitro. Such findings suggest that the cells in the iNCMSC-TEC retained their chondrogenic potential after the tissue engineering process (Figures 6(e) and 6(f)).

### 3.4. Implantation of iNCMSC-TEC into Osteochondral Defects

For the implantation trials, in addition to the untreated group, a group of hBMMSC-derived TEC (hBM-TEC) were included as a positive control to compare the efficacy of cell therapy. The hBMMSCs and hBM-TEC were cultured under the same conditions, and their MSC characteristics were confirmed by same protocols (Figure 3(a) and Supplementary Figure S3).

**Figure 7:**
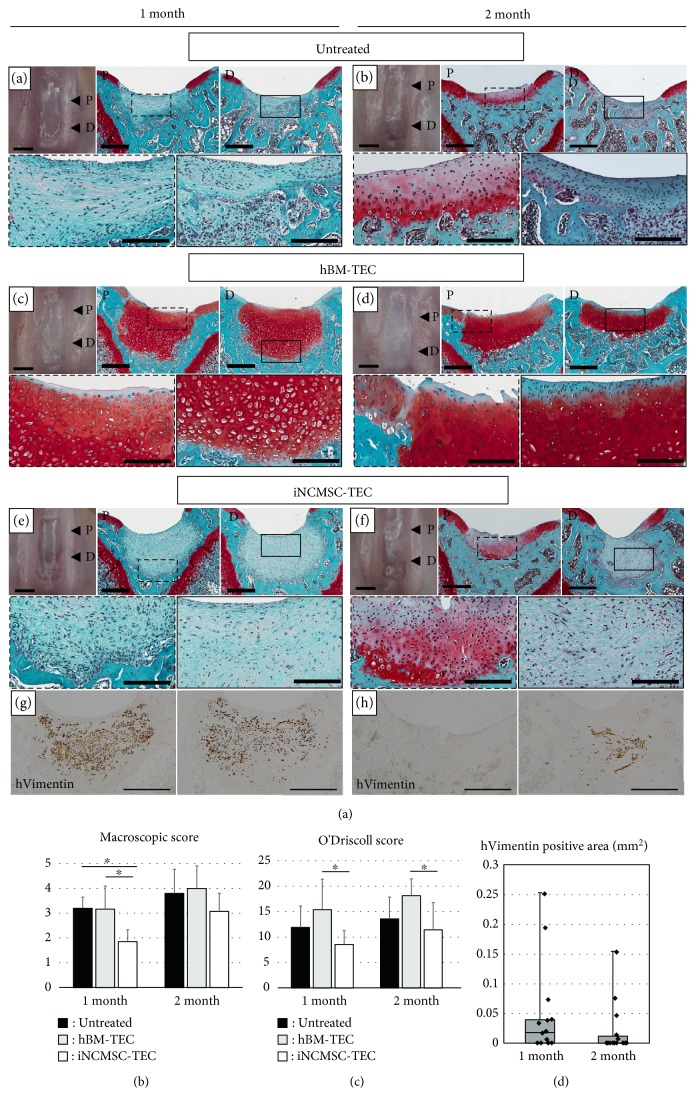
In vivo transplantation of TEC to rat osteochondral defects. (a–f) Macroappearance and Safranin-O staining (Saf-O) for each group at 1 month and 2 months. Inset boxes in the upper panels are magnified in lower panels with the same frame. Representative data are shown (*N* = 5 knees for the empty group, *N* = 6 knees for the hBM-TEC group, and *N* = 7 knees for the iNCMSC-TEC group at each endpoint). Histological assessments were conducted at two different points as shown in the macroimages (“P” represents “proximal section” and “D” presents “distal section”). Scale bars = 1 mm (macroimage), 500 *μ*m (upper panels of Saf-O), and 200 *μ*m (lower panels of Saf-O). (g, h) Human vimentin immunostaining of the iNCMSC-TEC group at 1 month and 2 months. Scale bars = 500 *μ*m. (i) Macroscopic score based on their gross appearance [57]. Averaged score with standard deviation is shown. ^∗^*P* < 0.05 by the Steel-Dwass test. (j) Histological grading by the O'Driscoll scoring system based on Saf-O staining using two specimens (“P” and “D”) per each knee. Data are expressed as the mean ± SD for each group. ^∗^*P* < 0.05 by the Steel-Dwass test. (k) The quantitation of transplanted iNCMSCs remaining at the implantation site was calculated from their hVimentin-DAB positive area. Data are shown as box-and-whisker plots and dot plots. Abbreviations: TEC: Tissue-engineered construct; hBM: Human bone marrow mesenchymal stem cell; iNCMSC: Induced neural crest-derived mesenchymal stem cells.

In the untreated group, the spontaneous repair of subchondral bone defects was apparent; however, the repair tissue was mainly composed of fibrous- or fibrocartilage-like tissues rich in type 1 collagen and which lacked staining for Safranin-O and type 2 collagen (Figures 7(a) and 7(b) and Supplementary Figure S4A). In the hBM-TEC group, macroscopic assessment showed that the defect was filled with white tissue. Histologically, this repair tissue stained strongly for Safranin-O and type 2 collagen, similar to hyaline cartilage (Figures 7(c) and 7(d) and Supplementary Figure S4B). The defects were fully filled with a hyaline-like cartilaginous tissue when assessed at 1-month postimplantation. Over time, subchondral bone formation proceeded further, which resulted in the upper migration of the osteochondral junction by 2 months postimplantation (Figure 7(d)).

**Figure 8:**
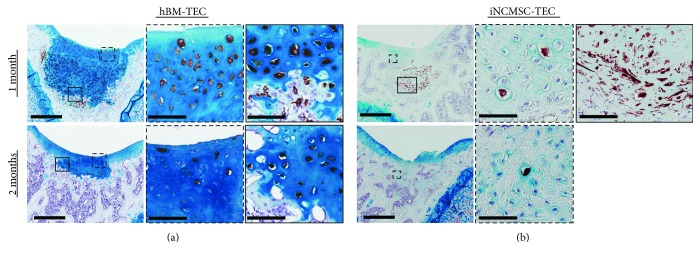
Vimentin and alcian blue staining of in vivo implanted TEC. (a, b) hVimentin/alcian blue staining for the hBM-TEC and iNCMSC-TEC groups at 1 month and 2 months. Right panels are magnification images of inset boxes of the left panels. Scale bars = 500 *μ*m and 50 *μ*m (high-magnified images). Abbreviations: TEC: Tissue-engineered construct; hBM: Human bone marrow-derived mesenchymal stem cells; iNCMSC: Induced neural crest-derived mesenchymal stem cells.

In the iNCMSC-TEC group, macroscopically, the defects were filled with transparent tissue at 1 month (Figure 7(e))s and partially filled with a white tissue at 2 months (Figure 7(f)). Histologically, the repair tissue was mostly composed of a fibrous tissue rich in type 1 collagen and which lacked staining for Safranin-O and type 2 collagen (Figures 7(e) and 7(f) and Supplementary Figure S4C). Notably, many implanted cells positive for human vimentin remained in the implantation site without specific differentiation toward the osteochondral lineage, and these appeared to inhibit the repair of subchondral bone (Figures 7(g) and 7(h)). Moreover, fibrous tissues, which were highly stained for COL1 immunostaining, were detected in such specimens (Supplementary Figure S4C black arrow). Based on the results of both the macroscopic and histological grading of repair tissue, the iNCMSC-TEC group exhibited the worst repair quality of the three groups assessed (Figures 7(i) and 7(j)). With regard to their potential residual tumorigenic capacity, the iNCMSCs did not detectably proliferate abnormally in vivo for the duration of the two-month follow-up (Figure 7(k)). Human vimentin positive cells were detected in 7 out of 7 knees and 10 out of 14 specimens tested at 1 month and in 5 out of 7 knees and 6 out of 14 specimens at 2 months. In addition, Ki-67, a proliferation marker used for the detection of malignant cells, was not detected in any specimens throughout the experiment (Supplementary Figure S5). Based on the combined staining for human vimentin and sGAG, it was concluded that implanted hBMMSCs differentiated toward the osteochondral lineage and formed abundant cartilaginous and bony tissue in response to the in vivo environment (Figure 8(a)). On the other hand, most of iNCMSCs were still detected in the repair tissue without showing any overt signs of differentiation toward the osteochondral lineages. However, a few iNCMSCs were detected in the area of the remodeled subchondral bone, and they appeared to exhibit a similar cell shape to the surrounding osteocytes (Figure 8(b)).

## 4. Discussion

In the present study, MSC-like cells were reproducibly generated from iNCCs (iNCMSCs) using a relatively simple protocol and characterized for their properties with a focus on the efficiency of MSC generation, their nontumorigenic phenotype, their in vitro differentiation capacities, and on their ability to repair osteochondral defects in vivo. It was determined that iNCMSCs and iNCMSC-TEC generated with passage 2 cells exhibited a capacity for chondrogenesis in vitro; nevertheless, they did not spontaneously undergo chondrogenesis in the in vivo environment after implantation into osteochondral defects, unlike the parallel studies using a human bone marrow-derived MSC-TEC. Our in vitro experiments showed that an effective dose of BMP2 was critical to promote chondrogenesis of iNCMSCs and the presence of serum very sensitively inhibited their chondrogenesis (see Figures 4(h) and 4(i)). Considering the fact that creation of an osteochondral defect is accompanied by a certain amount of bleeding, it would be reasonable to conclude that the alteration of biological environment by such bleeding, such as related to growth factor concentration and addition of serum-derived factors, could have negatively affected osteochondral differentiation of iNCMSCs at the site of the lesion.

With regard to the tumorigenesis of iNCMSC, no abnormal proliferation of iNCMSCs in vivo was observed for the two months following implantation, suggesting no involvement of residual malignant cells in the implanted TEC. The in vitro experiments showed that iNCMSCs exhibited high expandability in vitro for an initial 6-7 weeks, and then growth stopped by 8–10 weeks. During the culture duration, no evidence for the contamination of the MSC populations with residual PSCs or the emergence of malignant cells was observed. These observations are consistent with previous studies on the tumorigenesis of fetal or PSC-derived neural crest cells [81, 82]. Taken together, these findings suggest that iNCMSCs are safe and comparable to conventional MSCs in this regard.

Although, we could not confirm the in vivo realization of the in vitro potential of iNCMSCs to affect the repair of osteochondral lesions when implanted in an undifferentiated state, it is reasonable to consider generating a hyaline cartilaginous tissue ex vivo with cells differentiated towards chondrogenesis before implantation as the next step in the realization of this potential for tissue repair. Currently, direct differentiation toward chondrocytes from PSCs via mesodermal origin is being widely studied in efforts to generate cartilaginous tissue ex vivo [74, 83, 84]. However, such protocols require multiple serial procedures involving several different mediums and coating vessels. Due to the lack of a purification step, the risks for contamination by tumorigenic pluripotent cells [73, 85] and heterogenity with regard to the presence of cells of different lineage cells are a concern with such protocols. On the other hand, in mesodermal development, paraxial mesoderms, which are sorted by PDGFRa^(+)^/KDR^(−)^ cells from PSCs [22, 86–90], could be promising progeny to generate hyaline cartilage. These paraxial mesoderm-like cells show the best chondrogenic potential in comparison with other mesodermal progeny, but such cells do not emerge with high probability (10–50% based on FACS analysis). In addition, there are no current methods to maintain and/or expand them at any intermediate stages. In this regard, the CD271^high^-iNCCs used in this study can be obtained with high efficacy (70–90% based on FACS analysis) [30, 32, 43] and purified homogeneously after further maintenance culture, even without cell sorting [31]. Moreover, the present study showed that homogeneous mesenchymal progenitors could be simply induced from iNCCs without the need for additional cell sorting (see Figures 1 and 3). Based on the above discussion, and the characteristics of iNCMSC generation and safety, they could be a reliable cell source for the creation of ex vivo cartilaginous tissues.

However, for the effective implementation of this strategy, one would need to address several issues. Firstly, in the in vitro differentiation experiments, iNCMSCs exhibited features of hypertrophic chondrogenesis with type 10 collagen upregulation following exposure to the chondrogenic differentiation media. Secondly, the resultant chondrogenic pellets following exposure to the chondrogenic differentiation medium contained fibrous or necrotic tissue in their center. In order to overcome such limitations, some modifications to the current protocols will be required, not only for induction of chondrogenesis but also for the transition and maintenance of the MSC-like cells. In the current studies, a serum containing media was used for the induction and maintenance of the MSC-like cells from iNCCs. However, this approach may affect the phenotype of the MSCs regarding features which are different from developmental mesenchymal cells [39–42]. In other studies focusing on the generation of chondrogenic progeny via neural crest cells, Umeda et al. used TGF*β* stimulation for 72 hours prior to aggregate culture of iNCCs and succeeded in the formation of homogeneous hyaline cartilage-like tissues [31]. These authors reported that after further expansion of the cells in such TGF*β* containing medium, or in a serum medium, they lost their chondrogenic potential rapidly. However, in the present studies, the iNCCs retained this property until passage 3 after MSC induction with a serum medium. Thus, the initial steps and further maintenance of the iNCC-derived cells could drastically affect their phenotype. This was shown in the present study regarding alterations in CD90 expression with bFGF supplementation (see Figure 3(a) and Supplementary Figure S2). With further optimization of the protocols to induce mesenchymal progenitors and promote their chondrogenic capacity, iNCMSCs could be an attractive cell source for applications in regenerative therapy to repair osteochondral lesions.

## 5. Conclusion

The studies reported here demonstrate that a homogenous population of human MSC-like cells could be readily generated from iPSCs via the neural crest lineage. In vitro, these cells exhibited a capacity for chondrogenesis and osteogenesis that was comparable to human bone marrow-derived MSCs. However, the iNCMSC failed to realize the in vitro determined potential as they failed to affect regenerative repair of osteochondral defects in vivo. Furthermore, no residual tumorigenic responses were detected following implantation into osteochondral defects in vivo throughout a 2-month follow-up period. Based on the relative safety associated with the observed cell homogeneity and their high proliferative and osteochondral differentiation capacity, these iPS-derived MSCs could be an attractive source of cells for regenerative therapy applications for osteochondral repair. However, optimization of a local delivery system, as well as enhanced culture protocols for mesenchymal transition and differentiation, will be required to fulfill their potential for future therapeutic use in vivo.

## Supplementary Material

Table S1: FACS antibodies.
Table S2: IHC antibodies.
Table S3: List of TaqMan gene expresssion assays.
Figure S1: NC/MSC marker analysis for long passaged iNCMSCs (P4, P8).
Figure S2: iNCMSCs expanded without bFGF sustained CD90 expression.
Figure S3: The properties of human BM-MSCs in vitro.
Figure S4: Immunostaining for collagens.
Figure S5: Tumorigenesis of iNCMSCs after in vivo transplantation.
