# Supplementary material for: Characterization of Mesenchymal Stem Cell-Like Cells Derived From Human iPSCs via Neural Crest Development and Their Application for Osteochondral Repair
Source: Stem Cells Int. 2017 May 10;2017:1960965. doi: 10.1155/2017/1960965 (PMC5451770; doi:10.1155/2017/1960965)

***Supplementary Materials and Methods***

*Cell culture*

Human iPSCs (409B2) were provided by the RIKEN BRC through the National Bio-Resource Project of the MEXT, Japan. hiPSCs were cultured with mTeSR1 (veritas) and iMatrix (Nippi) coated vessels without feeder cells. For assessment of iNCMSCs, 409B2-hiPSCs were used as positive control to detect some PSCs specific markers.

KG-1, acute myeloid leukemia cell lines, was provided by the National Institutes of Biomedical Innovation, Health and Nutrition through JCBR Cell Bank, Japan. KG-1 was cultured in 20%FBS-IMDM (gibco), and used for positive control of hematopoietic stem cell markers.

Human bone marrow derived MSCs were purchased from LONZA (Catalog # PT-2501, Lot # 0000451491), and cultured in same condition to iNCMSCs. In brief, the medium was 10%FBS-aMEM supplemented with 5 ng/mL bFGF, and passaged with 0.25% Trypsin-EDTA. The cells from passage 3 were used for all assays.

Supplemental Table 1

| **FACS antibodies** | |  |  |
| --- | --- | --- | --- |
|  |  |  |  |
| **Target (antigen)** | **Conjugate** | **Company** | **Catalog No.** |
| CD271 | PE | BD | 557196 |
| CD44 | FITC | Biolegend | 338804 |
| CD73 | PE | Biolegend | 344004 |
| CD90 | FITC | Biolegend | 328108 |
| CD105 | APC | Biolegend | 323208 |
| CD34 | FITC | BD | 555821 |
| CD45 | PE | Biolegend | 304007 |
| TRA-1-60 | Alexa Fluor 488 | Biolegend | 330614 |
| rBC2LCN | FITC | Wako | 180-02991 |

Supplemental table 2

Supplemental table 3

***Supplementary Figure legends***

Fig.S1 **NC/MSC marker analysis for long passaged iNCMSCs (P4, P8)**

**(A)**: FACS analysis of neural crest and mesenchymal stem cells surface markers in iNCMSCs (P4, P8).

**(B)**: Positive controls of CD34 and CD45 using KG-1 which is acute myeloid leukemia cell lines.

Abbreviation: iNCMSCs, induced neural crest derived mesenchymal stem cells; BM-MSCs, bone marrow derived mesenchymal stem cells.

Fig.S2 **iNCMSCs expanded without bFGF sustained CD90 expression.**

**(A):** Cell morphology of iNCMSCs at P4 expanded with or without bFGF. Scale bars = 100 μm.

**(B)**: FACS analysis of CD90 of iNCMSCs at P4 expanded with or without bFGF.

**(C)**: CD90 positive cell rate during expansion with or without bFGF. Each symbol represents each passage.

Abbreviation: bFGF, basic fibroblast growth factor.

Fig.S3 **The properties of human BM-MSCs *in vitro*.**

**(A)**: Cell morphology of human BM-MSCs passaged twice with 10%FBS-aMEM, 5 ng/mL bFGF.

**(B)**: Oil red staining of human BM-MSCs cultured in adipogenesis medium at day 9.

**(C,D)**: Osteogenesis of human BM-MSCs cultured in osteogenesis medium. ALP staining at day 7 **(C)** and Alizarin Red S staining at day 14 **(D)**.

**(E)**: Saf-O staining of chondrogenic pellet cultured with TGFb3 and BMP2 at day 28.

**(F)**: The gross appearance of hBM-TEC developed in 24 well plate at day 7.

Scale bars = 100 μm **(A-D)** and 500 μm **(E)**.

Fig.S4 **Immunostaining for collagens.**

**(A-C)**: Immunostaining for COL1 and COL2 for each group at 1 month and 2 month.

Right panels were magnification image of black arrow of left panels. Scale bars = 500 μm and 100 μm (high magnified images.)

Abbreviations: COL1, type 1 collagen; COL2, type 2 collagen; TEC, tissue engineered construct; hBM, human bone marrow derived mesenchymal stem cells; iNCMSC, induced neural crest derived mesenchymal stem cells.

Fig.S5 **Tumorigenesis of iNCMSCs after *in vivo* transplantation.**

**(A)**: Histological analysis of iNCMSC-TEC transplanted knee at 2 month. HE staining and immunostaining (hVimentin and Ki-67) were performed with serial sections.

**(B)**: EB-outgrowth cells derived from 253G1-iPSCs were transplanted into osteochondral defect of nude rat. HE staining and Ki-67 immunostaining at 1 month specimen.

Scale bars = 500 μm (upper panels) and 100 μm (lower panels).

Abbreviation: iNCMSCs, induced neural crest derived mesenchymal stem cells; EB, embryoid body; HE, hematoxylin and eosin.

Fig.1S


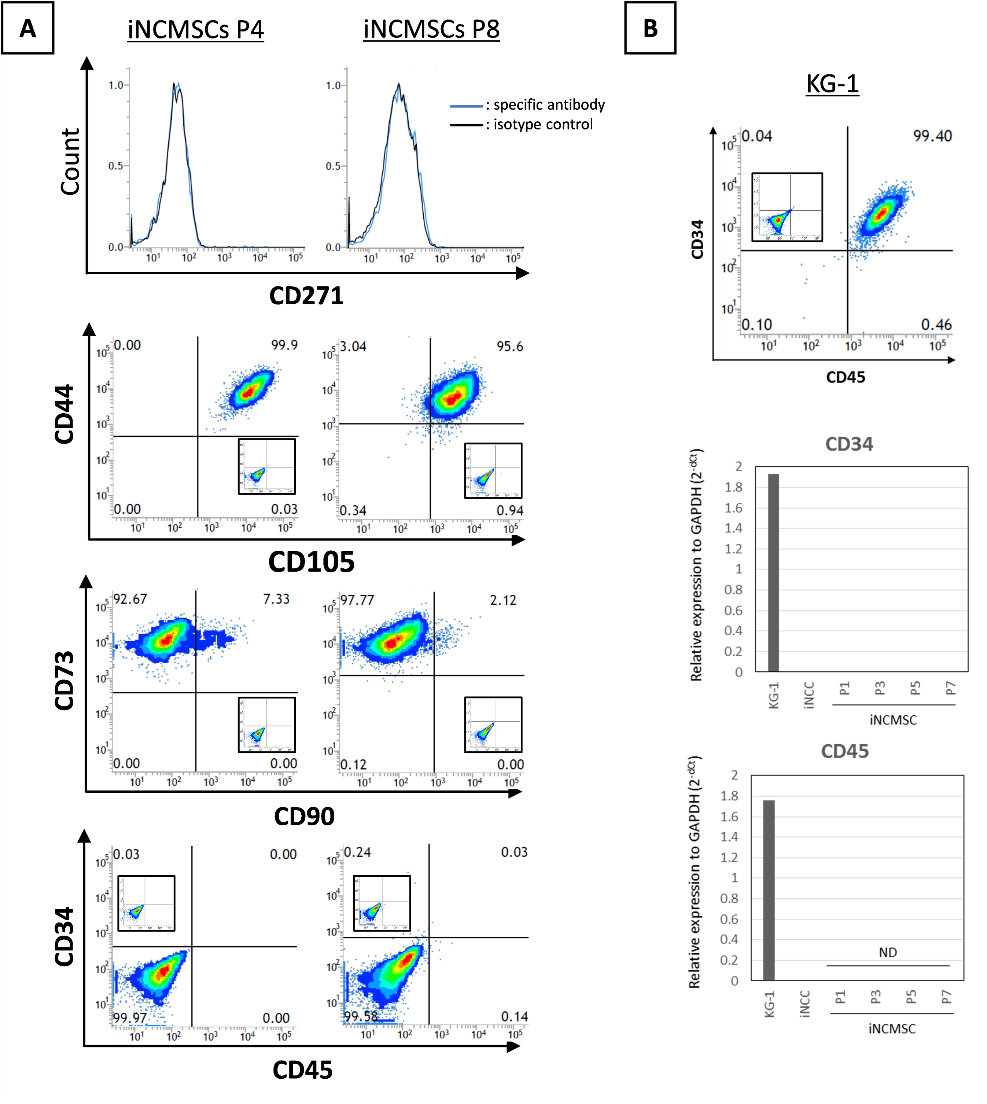


Fig.S2


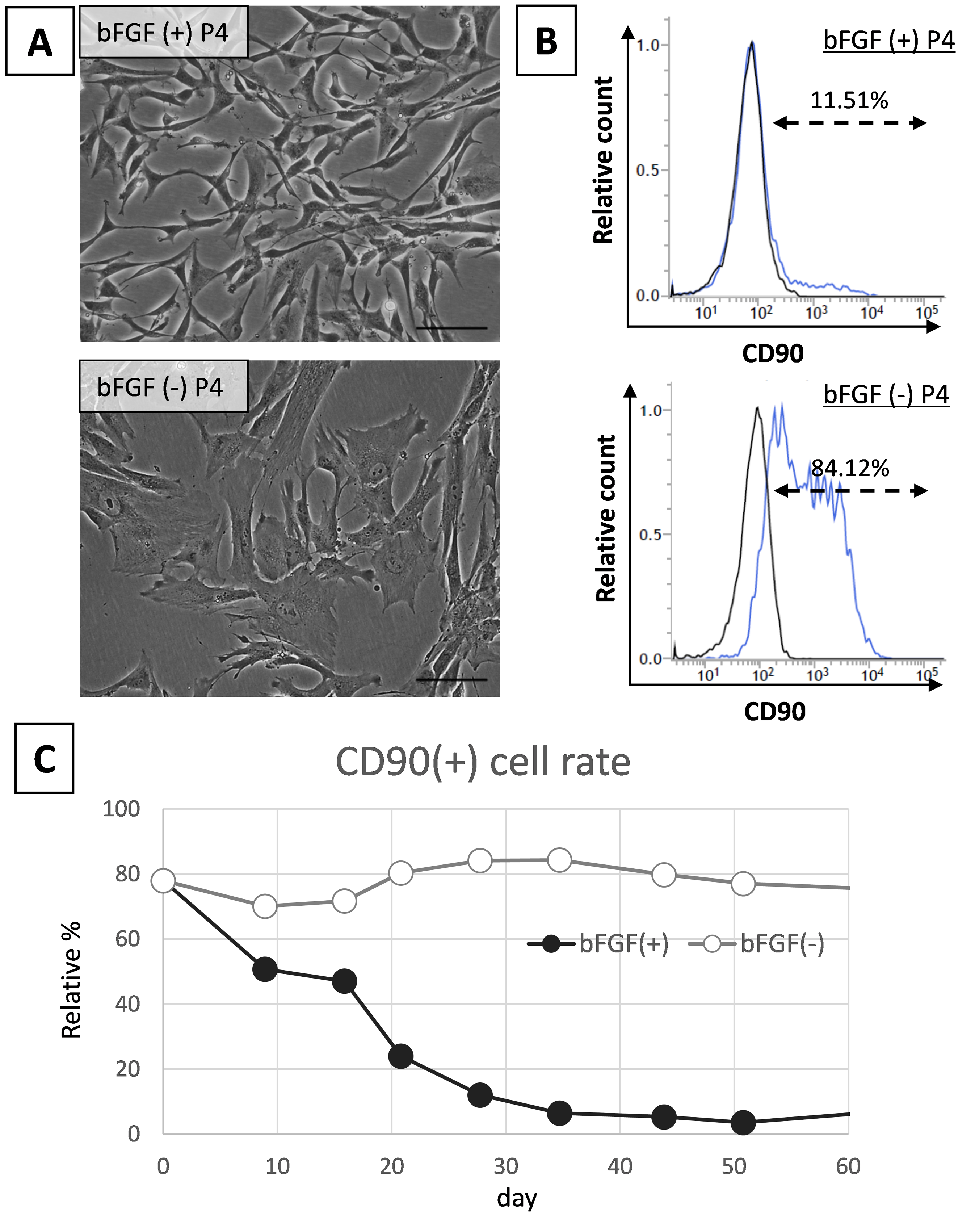


Fig.S3


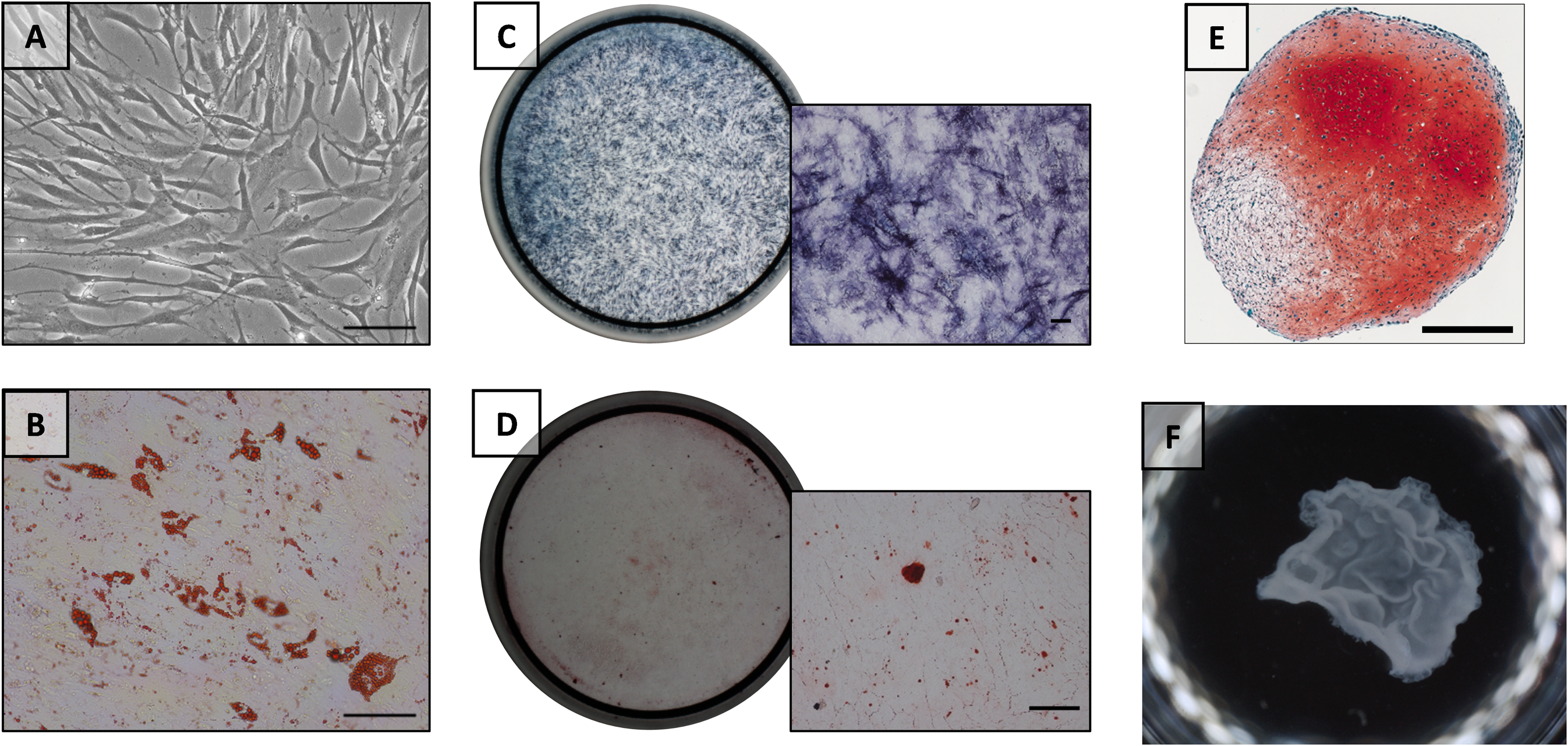


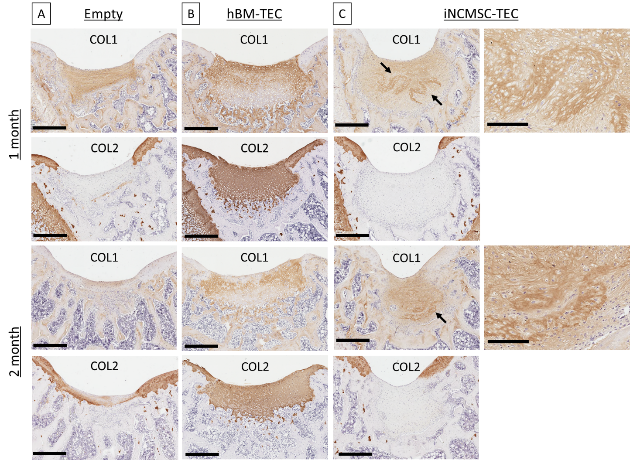
Fig.S4

Fig.S5


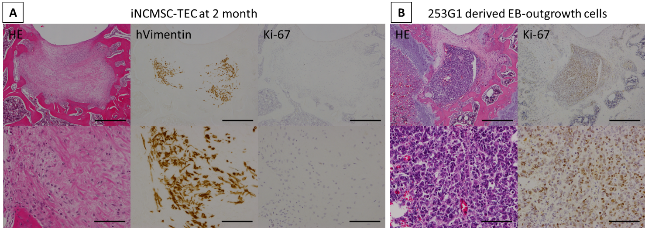

Supplement: Supplementary file 1 — Table S1: FACS antibodies. Table S2: IHC antibodies. Table S3: List of TaqMan gene expresssion assays. Figure S1: NC/MSC marker analysis for long passaged iNCMSCs (P4, P8). Figure S2: iNCMSCs expanded without bFGF sustained CD90 expression. Figure S3: The properties of human BM-MSCs in vitro. Figure S4: Immunostaining for collagens. Figure S5: Tumorigenesis of iNCMSCs after in vivo transplantation. [file 1960965.f1.docx]
