# Supplementary figures and images for: Characterization of Mesenchymal Stem Cell-Like Cells Derived From Human iPSCs via Neural Crest Development and Their Application for Osteochondral Repair
Source: Stem Cells Int. 2017 May 10;2017:1960965. doi: 10.1155/2017/1960965 (PMC5451770; doi:10.1155/2017/1960965)

## Slide 1
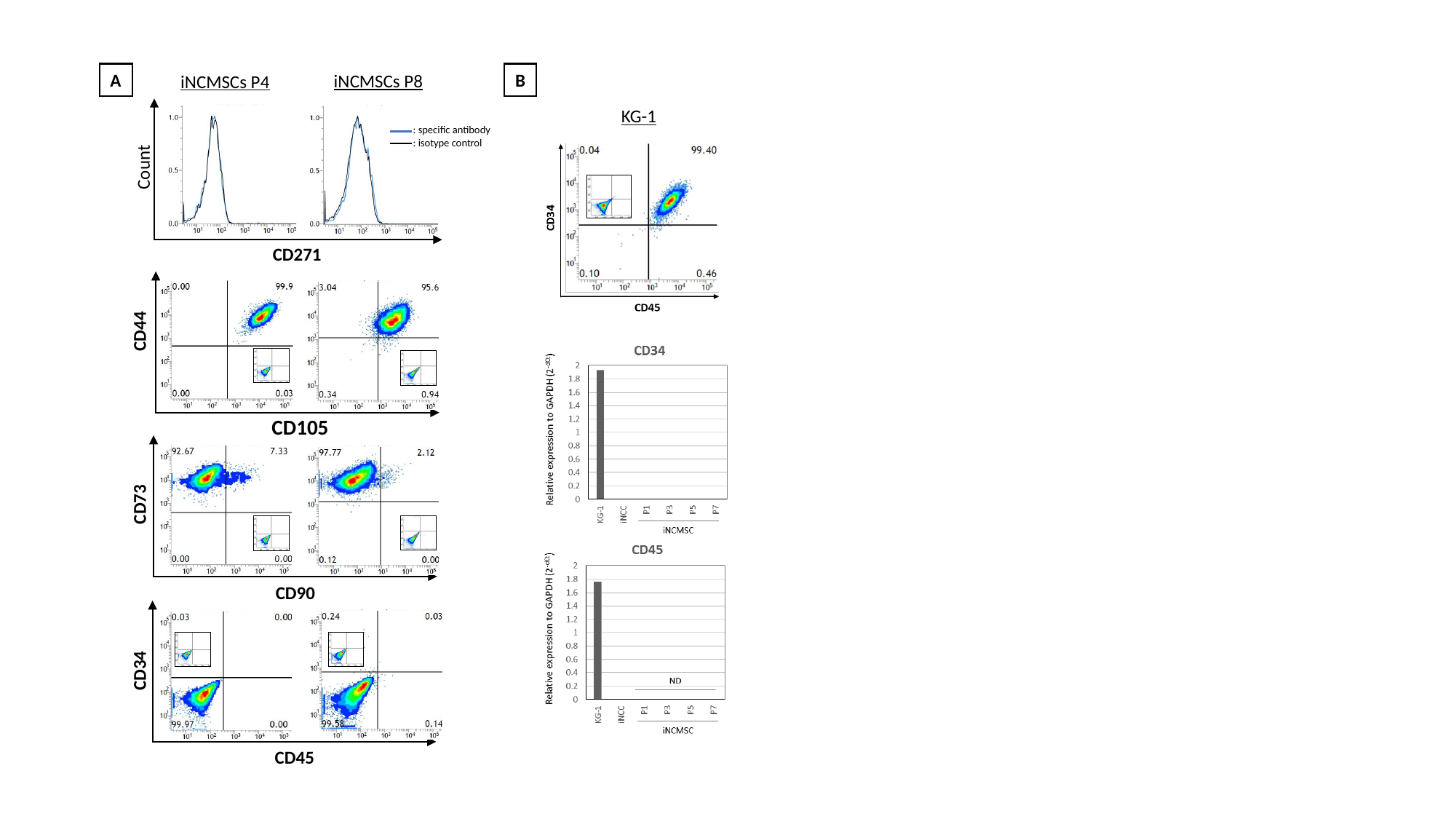

B
A
iNCMSCs P8
iNCMSCs P4
KG-1
: specific antibody
: isotype control
Count
CD271
CD44
CD105
CD73
CD90
CD34
CD45

Supplement: Supplementary file 5 [file 1960965.f5.pptx]

## Slide 1
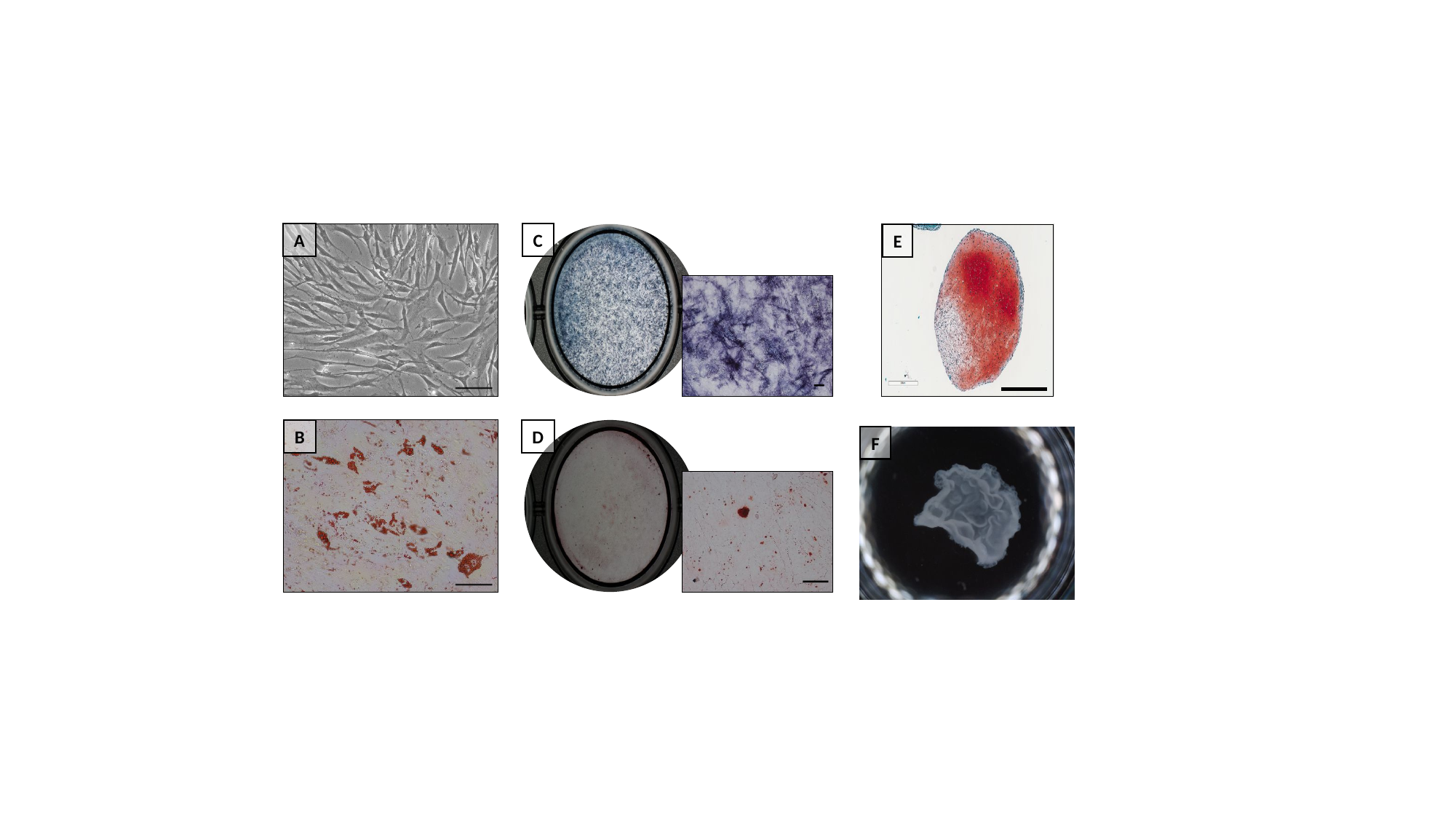

A
C
E
D
B
F

Supplement: Supplementary file 7 [file 1960965.f7.pptx]

## Slide 1
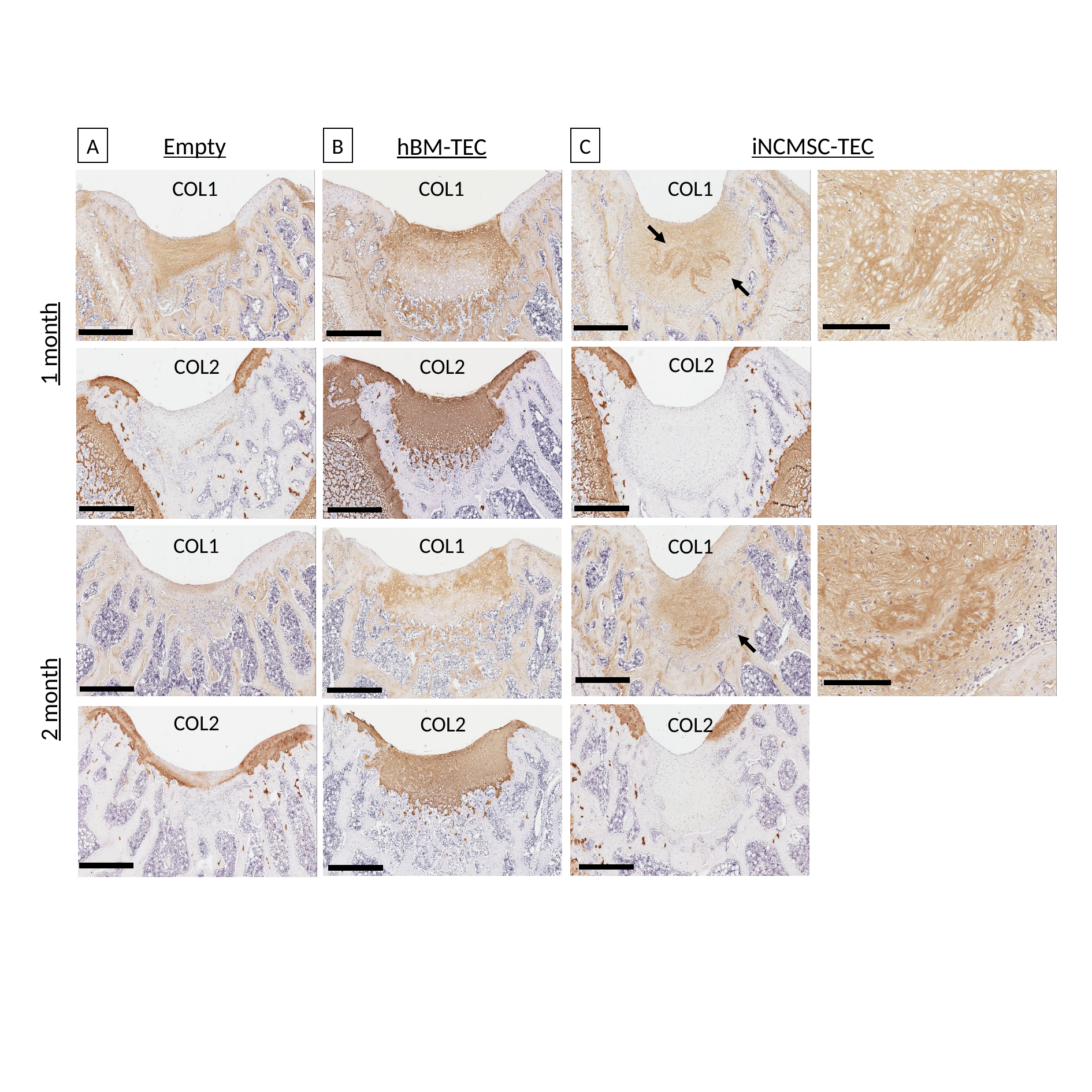

iNCMSC-TEC
Empty
hBM-TEC
A
B
C
COL1
COL1
COL1
1 month
COL2
COL2
COL2
COL1
COL1
COL1
2 month
COL2
COL2
COL2

Supplement: Supplementary file 8 [file 1960965.f8.pptx]

## Slide 1
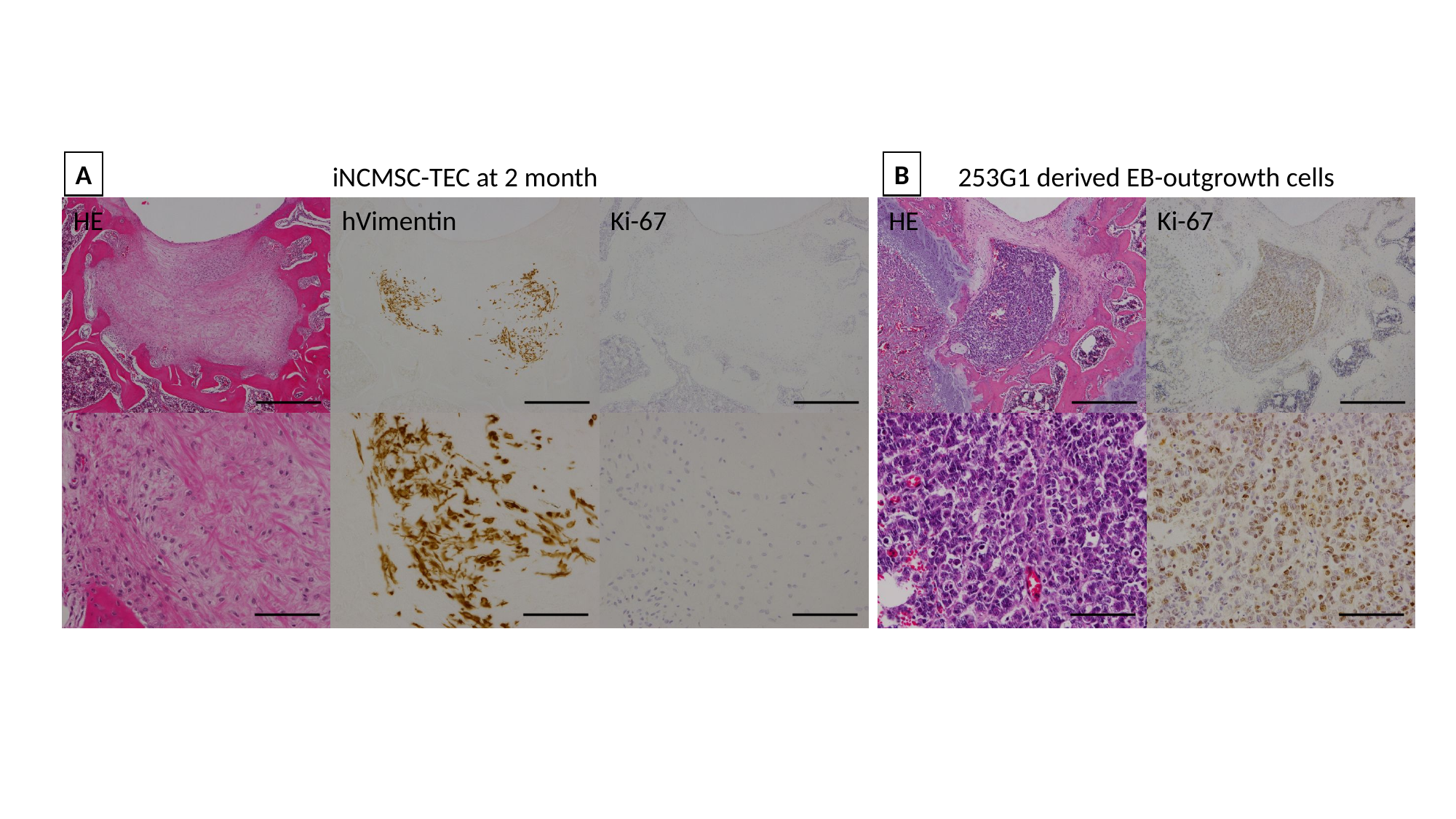

A
B
iNCMSC-TEC at 2 month
253G1 derived EB-outgrowth cells
HE
hVimentin
Ki-67
HE
Ki-67

Supplement: Supplementary file 9 [file 1960965.f9.pptx]
