# Supplementary material for: Characterization of Mesenchymal Stem Cell-Like Cells Derived From Human iPSCs via Neural Crest Development and Their Application for Osteochondral Repair
Source: Stem Cells Int. 2017 May 10;2017:1960965. doi: 10.1155/2017/1960965 (PMC5451770; doi:10.1155/2017/1960965)
Supplement: Supplementary file 6 [file 1960965.f6.pptx]

## Slide 1
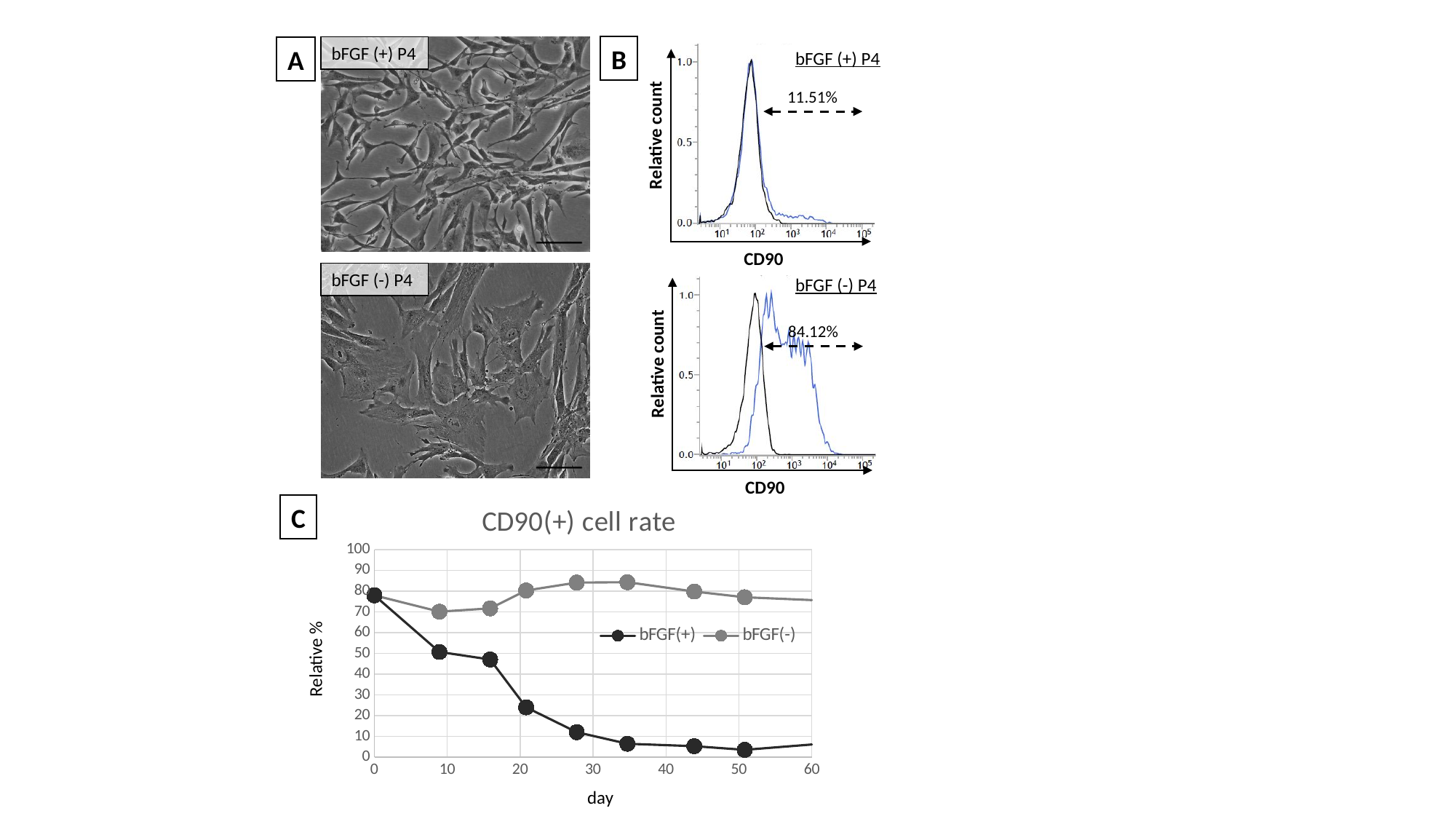

bFGF (+) P4
B
A
bFGF (+) P4
11.51%
Relative count
CD90
bFGF (-) P4
bFGF (-) P4
84.12%
Relative count
CD90
### Chart: CD90(+) cell rate
| Category | bFGF(+) | bFGF(-) |
|---|---|---|C
Relative %
day
